# Evolution of Bacterial Consortia in Spontaneously Started Rye Sourdoughs during Two Months of Daily Propagation

**DOI:** 10.1371/journal.pone.0095449

**Published:** 2014-04-18

**Authors:** Marianna Bessmeltseva, Ene Viiard, Jaak Simm, Toomas Paalme, Inga Sarand

**Affiliations:** 1 Competence Center of Food and Fermentation Technologies, Tallinn, Estonia; 2 Department of Food Processing, Tallinn University of Technology, Tallinn, Estonia; 3 Centre for Biology of Integrated Systems, Tallinn University of Technology, Tallinn, Estonia; 4 Department of Gene Technology, Tallinn University of Technology, Tallinn, Estonia; J. Craig Venter Institute, United States of America

## Abstract

The evolution of bacterial consortia was studied in six semi-solid rye sourdoughs during long-term backslopping at different temperatures. Each rye sourdough was started spontaneously in a laboratory (dough yield 200), propagated at either 20°C or 30°C, and renewed daily at an inoculation rate of 1∶10 for 56 days. The changes in bacterial diversity over time were followed by both DGGE coupled with partial 16S rRNA gene sequencing and pyrosequencing of bar-coded 16S rRNA gene amplicons. Four species from the genus *Lactobacillus (brevis, crustorum, plantarum,* and *paralimentarius)* were detected in different combinations in all sourdoughs after 56 propagation cycles. Facultative heterofermentative lactic acid bacteria dominated in sourdoughs fermented at 30°C, while both obligate and facultative heterofermentative LAB were found to dominate in sourdoughs fermented at 20°C. After 56 propagation cycles, *Kazachstania unispora* (formerly *Saccharomyces unisporus*) was identified as the only yeast species that dominated in sourdoughs fermented at 20°C, while different combinations of strains from four yeast species (*Kazachstania unispora*, *Saccharomyces cerevisiae*, *Candida krusei* and *Candida glabrata)* were detected in sourdoughs propagated at 30°C. The evolution of bacterial communities in sourdoughs fermented at the same temperature did not follow the same time course and changes in the composition of dominant and subdominant bacterial communities occurred even after six weeks of backslopping.

## Introduction

Sourdough is a mixture of flour and water fermented with a microbial community mainly consisting of lactic acid bacteria (LAB) and yeasts. LAB dominate the microbial community and are responsible for acid production while yeasts work to leaven the dough [1], [2]. Lactic acid fermentation plays an important role in the production of rye bread by both decreasing the activity of α-amylase and improving dough texture [3].

Lactobacillus brevis, Lactobacillus plantarum, and Lactobacillus sanfranciscensis are the LAB species most frequently isolated from rye sourdoughs [2], [4]–[5]. However, Lactobacillus amylovorus, Lactobacillus fermentum, Lactobacillus helveticus, Lactobacillus panis, Lactobacillus pontis, and Lactobacillus reuteri have also been identified as dominant species in rye sourdoughs [4], [6]–[9]. Since rye flours have a generally higher extraction rate than wheat flours [5] rye sourdoughs are characterized by higher buffering capacity compared with wheat and spelt sourdoughs but also higher concentration of mannitol and amino acids [8], [10]. Despite this, the same LAB species are able to dominate the microbial communities within mature sourdoughs made with different types of flour under otherwise identical conditions [8], [10]–[11]. Recently, it has been shown that initial differences in the microbial communities found in spontaneously started rye and wheat sourdoughs decreases during backslopping propagations and that a common core microbiota is established [11].

The establishment and stability of microbial consortia in sourdoughs depends on the microbial communities within the raw materials, the chemical composition of the raw materials, and interactions between the microorganisms, together with fermentation parameters such as temperature, inoculum size, dough yield, and fermentation length [12]–[16]. Previous studies on the dynamics of microbial communities in spontaneously started sourdoughs have stated that the establishment of a stable consortium occurs through a three-stage evolution process within a few days during which one may observe the prevalence of sourdough-atypical LAB, sourdough-typical LAB, and highly adapted sourdough-typical LAB [2], [8], [10]–[11]. Most of these studies have been carried out with liquid sourdoughs fermented using continuous stirring and propagated over a short period of time (maximum two weeks). Traditional Estonian rye sourdoughs can be classified as semi-solid because they have a relatively dense consistency. They are fermented at ambient temperature, which may vary between 18–27°C depending on the season. Because the water content (dough yield) and fermentation temperature are the main factors that affect the composition of the bacterial community [2], we evaluated the establishment of microbial consortia in spontaneously fermented rye sourdoughs with a low dough yield at two different temperatures (20 and 30°C) backslopped daily for 56 days. The aims of our study are (i) to gain insight into the stability of bacteria communities after their initial establishment in mature sourdough, (ii) to determine the effect of fermentation temperature on the development of bacterial communities in sourdoughs, (iii) to assess the reproducibility of the development of microbial communities in sourdough when applying fermentation conditions with low dough yield.

## Materials and Methods

### Sourdough Fermentation and Sampling

Sourdough fermentation was initiated in six 400 ml sterile Stomacher circulator bags (Seward Limited, England) by mixing 150 g of rye flour and 150 g of sterile 0.5% NaCl solution. Rye flour (Type 1370, Tartu Mill, Estonia) from a single 50 kg bag was used during the entire experiment. Each sourdough was mixed for 15 minutes at 100 rpm using a Stomacher 400 circulator (Seward Limited, England), the bags were sealed with tape, and positioned vertically into an incubator. Three sourdough batches were fermented at 20°C and another three at 30°C. After 24 hours of fermentation, each sourdough was thoroughly mixed in the circulator for 5 min at 100 rpm and renewed at an inoculation rate of 1∶10 by mixing 30 g of sourdough, 135 g of sterile 0.5% NaCl solution, and 135 g of rye flour. In total, each sourdough experienced 56 backslopping cycles. The three sourdoughs fermented at 20°C are referred to as 20-I, 20-II, and 20-III while those fermented at 30°C are referred to as 30-I, 30-II, and 30-III. Day 0 indicates the start of the experiment.

Both the acidity, in units of pH, and total titratable acidity (TTA) were measured using a DL22 Food and Beverage Analyzer (Mettler-Toledo LLC., USA) at the end of each fermentation cycle prior to renewal. Following this, five grams of sourdough was homogenized with 50 ml of distilled water using a Polytron PT2100 homogenizer (Kinematica AG, Switzerland).

Both bacterial plate counts and DGGE analysis were carried out on days 0, 1, 3, 5, 7, 10, 15, 21, 28, 35, 42, and 56 of the experiment. Pyrosequencing was performed on samples from days 1, 3, 5, 7, 21, and 56. The sample from day 0 was taken from the rye flour and water mixture immediately after mixing.

### Isolation and Characterization of Lactic Acid Bacteria and Yeasts

Five grams of sourdough were supplemented with sterile 0.85% NaCl solution up to a volume of 50 ml. The mixture was then homogenized by vortexing. Decimal dilutions were plated on to both sourdough bacteria (SDB) agar (maltose, 2.0%; yeast extract, 1%; Tween 80, 0.03%, trypticase 0.6%; pH 5.6) and de Man, Rogosa and Sharpe (MRS) agar (Lab M Ltd, UK) with 100 µg/ml cycloheximide (Sigma-Aldrich, USA).

The plates were incubated at the same temperature the sourdough was fermented at (20 or 30°C). Incubation was carried out for 48 h under anaerobic conditions (AnaeroGen, Oxoid). Colony forming units (CFU) were counted from the agar media using suitable dilutions.

For each of the six sourdough samples collected on day 56, 20 colonies were picked from the MRS and SDB agar plates (ten from each medium) for further analysis by rep-PCR. Colony picking was performed in succession from one sector of the plate. On day 56 samples were also plated on Yeast Extract Peptone Dextrose (YPD) agar (dextrose, 2.0%; peptone 2%; yeast extract, 1%) with 100 µg/ml chloramphenicol (Sigma-Aldrich, USA) and incubated at the same temperature the sample was fermented at (20 or 30°C). Ten colonies per sample were picked in succession from YPD agar plates and analyzed using RAPD-PCR.

### Extraction of Whole Genomic DNA

Total DNA extraction was performed using 5 g of sourdough, which had been homogenized by vortexing with 45 ml of sterile physiological solution [10]. This suspension was then centrifuged at 4°C for 5 minutes at 1000×g. The supernatant was collected and centrifuged at 4°C for 15 minutes at 5000×g. Each extraction of whole DNA was performed using a GenElute Bacterial Genomic DNA Kit (Sigma-Aldrich., USA), according to the manufacturer’s protocol. Total DNA was also extracted from the rye flour and water mixture immediately after mixing on day 0.

### Denaturing Gradient Gel Electrophoresis Analysis

The V3 region of the 16S rRNA gene was amplified from the whole genomic DNA using universal primers F357-GC and 518R as described in [10]. DGGE was carried out using the INGENYphorU system (Ingeny International Bv., Netherlands) as described in Viiard et al. [9]. All clearly visible bands were cut from the gel and incubated in TE buffer (10 mM Tris pH 7.5 and 1 mM EDTA pH 8.0) at 37°C for 1 h. Eluted DNA was reamplified using primers F357 and 518R and sequenced in a commercial facility (Estonian Biocentre, Tartu, Estonia). Nucleotide sequences were analyzed using the BLASTn algorithm together with the GenBank database (National Center for Biotechnology Information, USA).

### Pyrosequencing of Bar-coded 16S rRNA Gene Amplicons

Universal primers 8F and 357R were used for PCR amplification of the V1–V2 hypervariable regions of 16S rRNA genes [17]. The amplicon mixtures were pyrosequenced using a 454 GS FLX+ System (Roche 454 Life Sciences, USA) in a university facility (Centre for Biology of Integrated Systems, Estonia).

The resulting pyrosequencing data was analyzed using the software package MOTHUR, version 1.27.0 [18]. Reads shorter than 150 bps were removed from the dataset and the PyroNoise algorithm was used to discard both homopolymer-derived and PCR errors. The remaining sequences were aligned to the SILVA reference 16S ribosomal RNA database [19]. Chimeric sequences were filtered using the UChime method by applying the ‘chimera.uchime’ procedure in MOTHUR in *de novo* mode, which checks chimeras in each group separately. Operational Taxonomic Units (OTUs) were defined using the average neighbour clustering algorithm within MOTHUR with a 97% similarity threshold. Rarefaction curves and normalized OTU counts at 500 sequences were calculated using the R software package “vegan” version 2.0-7. In addition, we calculated the rate of forming new OTUs when one sequence is added to the set of 500 sequences. The closest match on the species level was found for each OTU using the BLASTn algorithm together with the GenBank database (National Center for Biotechnology Information, USA) with the parameters of 97% similarity and 90% coverage. The relative abundance of OTUs was calculated as the number of sequences for each OTU divided by the total number of bacterial sequences obtained for each sourdough sample.

### Statistical Analysis

Plate count data was subjected to Z-tests to both compare the results obtained at 20 and 30°C and to compare results obtained using different media. We tested the hypotheses that the difference between the results obtained at different conditions come from a distribution with mean zero. The nuisance parameter of the z-test is the sum of the standard deviation of the differences between the values obtained at two conditions and the standard deviations of the values obtained at each condition.

Acidity and TTA measurements obtained at 20 and 30°C were compared using a simple two sample Student’s t-test for samples with equal variance. The values tested were considered to come from distributions with different mean values when the p-value was below 0.05.

### DNA Isolation from Colonies, Rep-PCR Fingerprinting and Partial Sequencing of rRna Genes

Bacterial DNA was extracted from isolated colonies using Whatman indicating FTA MiniCards (GE Healthcare Ltd., UK) using a method provided by the manufacturer. Rep-PCR with primer (GTG)_5_ followed by agarose gel electrophoresis was performed as described by Viiard et al. [9]. Extraction of yeast DNA was carried out using a PureLink Genomic DNA mini Kit (Invitrogen, USA) using a method provided by the manufacturer. RAPD-PCR with an M13 primer was performed according to Andrighetto et al. [20]. Each fingerprint type was calculated as the ratio of similar fingerprints to the number of colonies analyzed. One or two representatives from each PCR fingerprint group were subjected to Sanger sequencing. The resulting 16S rRNA gene fragments were amplified using universal primers 27f-YM [21] and 16R1522 [22]. Yeasts were identified using standard protocols by amplifying the D1/D2 variable domains of the 26S rRNA gene with primer pair NL1 and NL4 [23]. Amplified fragments were purified with a GeneJET PCR Purification kit (Fermentas, Vilnius, Lithuania). Sequencing of the fragments was conducted at a commercial facility (Estonian Biocenter, Estonia). The resulting gene sequences were compared with the GenBank database using the BLASTn algorithm (National Center for Biotechnology Information, USA).

### Evaluation of Carbohydrate Fermentation Profiles

Carbohydrate fermentation profiles of selected LAB and yeast strains were determined with the identification kits API 50 CH and API 20 C AUX using methods provided by the manufacturer (bioMérieux, France).

## Results

### pH, Total Titratable Acidity, and Bacterial Plate Counts

Spontaneous rye sourdoughs were started at 20°C and 30°C in three parallels. After 24 hours of fermentation at 20°C the average viable count of bacteria increased from 2.0×10^5^ CFU/g in the raw flour to 5.7×10^8^ while the first fermentation cycle at 30°C resulted in 7.2×10^8^ CFU/g (Table 1). After the third backslopping cycle (day 3) the viable count of bacteria in all sourdoughs exceeded 10^9^ CFU/g. No significant (p<0.05) difference was found in the plate counts obtained from SDB or MRS media.

**Table 1:** Enumeration of lactic acid bacteria in spontaneous rye sourdoughs during two months of backslopping; viable counts are given as log CFU/g obtained on SDB and MRS media.

	Sourdoughs fermented at 20°C								Sourdoughs fermented at 30°C							
	SDB				MRS				SDB				MRS			
Day	20-I	20-II	20-III	Averageat 20°C	20-I	20-II	20-III	Average at 20°C	30-I	30-II	30-III	Averageat 30°C	30-I	30-II	30-III	Averageat 30°C
0	5.30	5.29	5.32	5.30±0.02	5.31	5.28	5.28	5.29±0.02	5.32	5.30	5.28	5.30±0.02	5.30	5.31	5.30	5.31±0.01
1	8.82	8.70	8.77	8.76±0.06	8.81	8.69	8.76	8.75±0.06	8.82	8.91	8.88	8.87±0.05	8.81	8.91	8.79	8.84±0.06
3	9.35	9.38	9.34	9.36±0.02*	9.28	9.24	9.26	9.26±0.02*	9.10	9.11	9.17	9.13±0.04*	9.05	9.13	9.07	9.08±0.04*
5	9.44	9.49	9.22	9.38±0.14	9.36	9.40	9.28	9.35±0.07*	9.30	9.39	9.34	9.34±0.05	9.17	9.16	9.07	9.13±0.05*
7	9.17	9.02	8.92	9.04±0.13	9.11	8.92	8.88	8.97±0.13	9.12	8.70	8.75	8.86±0.23	9.07	8.96	8.93	8.99±0.08
10	9.12	9.39	9.11	9.21±0.16	9.24	9.27	9.37	9.29±0.06	9.43	9.33	9.33	9.36±0.06	9.42	9.26	9.37	9.35±0.09
15	9.46	9.37	9.55	9.46±0.09	9.60	9.56	9.58	9.58±0.02*	9.44	9.30	9.36	9.37±0.07	9.30	9.33	9.32	9.32±0.02*
21	9.47	9.33	9.36	9.39±0.07	9.40	9.25	9.28	9.31±0.08	9.36	9.03	9.40	9.26±0.20	9.30	9.01	9.39	9.24±0.20
28	9.33	9.17	9.46	9.32±0.15	9.30	9.15	9.39	9.28±0.12	9.33	9.03	9.38	9.25±0.19	9.40	9.17	9.35	9.31±0.12
42	9.24	9.35	9.29	9.29±0.06	9.45	9.22	9.25	9.31±0.12	9.16	9.20	9.35	9.24±0.10	9.26	9.19	9.35	9.27±0.08
56	9.39	9.41	9.45	9.42±0.03*	9.48	9.35	9.49	9.44±0.08*	9.28	9.32	9.28	9.29±0.02*	9.31	9.26	9.21	9.26±0.05*

*Average values were statistically different between 20°C and 30°C fermentations according to Z-test.

**Figure 1:**
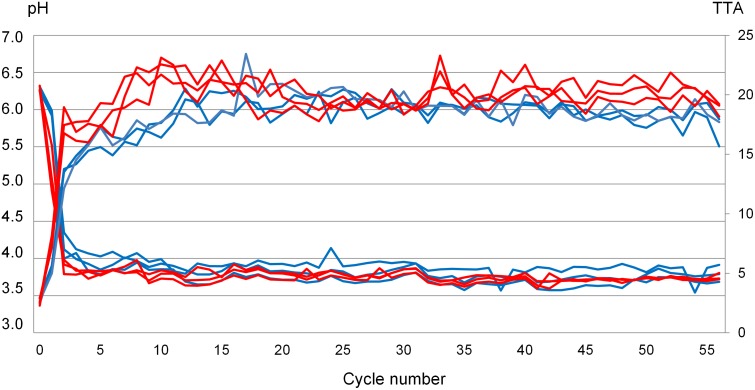
pH and total titratable acidity of six rye sourdoughs propagated at 20°C (20-I, 20-II and 20-III, shown in blue) and 30°C (30-I, 30-II and 30-III, shown in red) during 56 days.

Fermentation both increased the concentration of acids and lowered pH of the rye sourdoughs. For the fermentation series conducted at 30°C, the maximum TTA value reached (22.5±0.7) occurred on day 10 while the 20°C fermentation series displayed its maximum TTA value (19.5±1.0) on days 11 and 12 (Figure 1). The maximum value for the 30°C series was larger, however, the difference in TTA between sourdoughs fermented at different temperatures diminished during later propagation cycles (Figure 1).

During the first ten cycles, the pH in the sourdoughs fermented at 30°C decreased significantly more compared with those fermented at 20°C (Figure 1). However, during the later stages of backslopping, the acidity did not significantly differ between sourdoughs.

### Dynamics of the Bacterial Communities Determined by DGGE of 16S rRNA Gene PCR Amplicons

**Figure 2:**
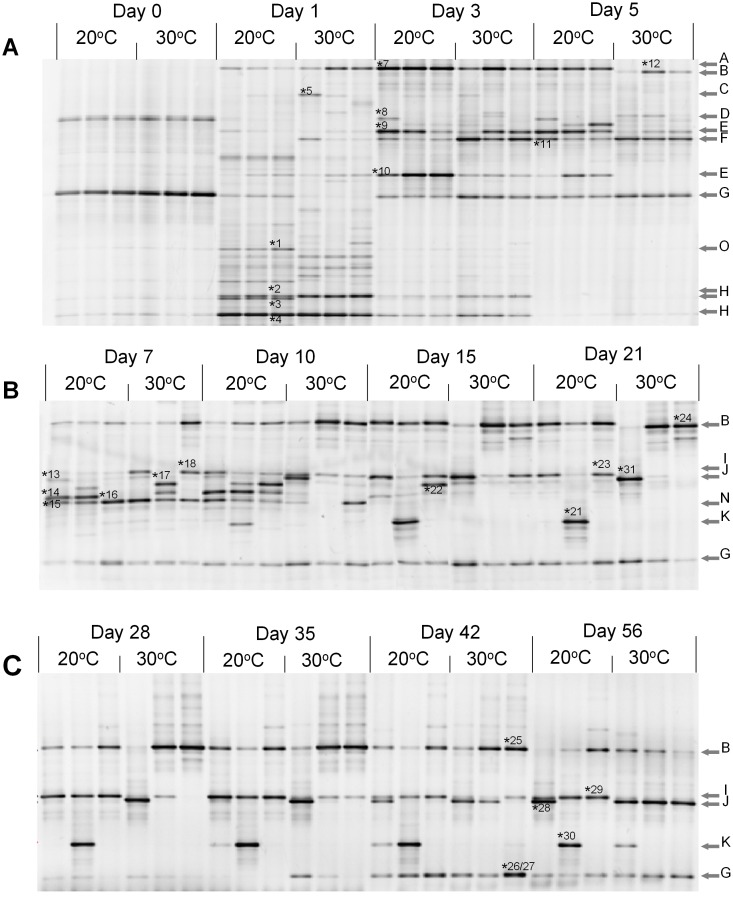
DGGE analysis of six rye sourdoughs propagated at 20°C (20-I, 20-II and 20-III) and 30°C (30-I, 30-II and 30-III) during 56 days. The arrows indicate specific bands for the following groups: (A) *Weissella* sp., (B) *Lactobacillus plantarum*, (C) *Enterobacteriaceae*, (D) *Lactobacillus* sp., (E) *Lactobacillus* sp., (F) *Pediococcus* sp., (G) Cereal chloroplast, (H) *Pantoea agglomerans*, (I) *Lactobacillus brevis*, (J) *Lactobacillus paralimentarius*, (K) *Lactobacillus crustorum*, (M) *Lactobacillus curvatus/graminis/sakei*, (N) *Pediococcus pentosaceus*, (O) *Gamma Proteobacteria* species. The bands marked by numbers were cut and sequenced. The closest matches are shown in the Table 2. A. DNA samples from days 0, 1, 3 and 5 of backslopping. B. DNA samples from days 7, 10, 15 and 21 of backslopping. C. DNA samples from days 28, 35, 42 and 56 of backslopping.

The highest diversity of species detected using DGGE analysis occurred after the first 24 hours of fermentation (Figure 2A). Over 15 bands were visible, however, only few of the sequences obtained by cutting these bands were identified at a similarity level ≥97% (Table 2). Of those identified, three belong to *Pantoea agglomerans,* one to a *Gamma Proteobacteria* species and one to a *Weissella* species. All of the bands identified were observed at both fermentation temperatures. After the third backslopping, the DGGE patterns of all six sourdoughs were similar to each other and consisted of fragments specific to the LAB genera *Weissella*, *Lactobacillus,* and *Pediococcus*. On day 5 the DGGE patterns of sourdoughs fermented at 20°C had not drastically changed. However, from sourdoughs fermented at 30°C, the bands specific to *Weissella* sp. and *Lactobacillus* sp. had disappeared and another band, identified as *Lactobacillus plantarum,* emerged (Figure 2A).

**Table 2:** Identification of DGGE bands obtained from six rye sourdoughs propagated at 20°C and 30°C during 56 days.

Band number onDGGE	Closest match inGenBank	Similarity	Accession Nr.
1	*Gamma Proteobacteria* species	163/168 (97%)	gb|GU352675.1
2	*Pantoea agglomerans*	166/166 (100%)	gb|KC355300.1
3	*Pantoea agglomerans*	165/166 (99%)	gb|KC355300.1
4	*Pantoea agglomerans*	165/166 (99%)	gb|KC355300.1
5	*Enterococcus casseliflavus*	120/140 (86%)	ref|NR_102793.1
7	*Weissella confusa*	160/168 (95%)	gb|KC845208.1
8	*Lactobacillus graminis*	97/106 (92%)	gb|KC836565.1
9	*Lactobacillus curvatus*	133/155 (86%)	gb|FJ609221.1
10	*Lactobacillus curvatus*	143/155 (92%)	gb|FJ609221.1
11	*Pediococcus acidilactici*	118/124 (95%)	gb|JF268323.1
12	*Lactobacillus plantarum*	138/138 (100%)	gb|JN863682.1
13	*Lactobacillus curvatus*	247/250 (99%)	gb|KF411435.1
13	*Lactobacillus graminis*	247/250 (99%)	gb|KF149819.1
13	*Lactobacillus sakei*	247/250 (99%)	gb|KF149680.1
14	*Lactobacillus curvatus*	206/217 (95%)	gb|KF411435.1
14	*Lactobacillus graminis*	206/217 (95%)	gb|KF149819.1
14	*Lactobacillus sakei*	206/217 (95%)	gb|KF149680.1
15	*Pediococcus pentosaceus*	276/276 (100%)	gb|JN851781.1
16	*Pediococcus pentosaceus*	254/257 (99%)	gb|JN851779.1
17	*Lactobacillus sakei*	276/276 (100%)	gb|JN851763.1
18	*Lactobacillus brevis*	257/257 (100%)	gb|JN863690.1
21	*Lactobacillus crustorum*	258/261 (99%)	gb|KF193907.1
23	*Lactobacillus brevis*	132/132 (100%)	gb|KC845206.1
24	*Lactobacillus plantarum*	140/140 (100%)	gb|JN863682.1
25	*Lactobacillus plantarum*	259/259 (100%)	gb|KF318862.1
26	*Secale cereale, complete genome*	121/122 (99%)	gb|KC912691.1
27	*Secale cereale, complete genome*	120/122 (99%)	gb|KC912691.1
28	*Lactobacillus paralimentarius*	113/113 (100%)	gb|KC755102.1
29	*Lactobacillus brevis*	129/129 (100%)	gb|KC845206.1
30	*Lactobacillus crustorum*	113/113 (100%)	gb|KC755094.1
31	*Lactobacillus paralimentarius*	114/114 (100%)	gb|KC755102.1

On day 7, a band specific to *L. plantarum* was also detected in all sourdoughs fermented at 20°C together with another band identified as *Pediococcus pentosaceus.* In addition, a third new band identified as *Lactobacillus curvatus/graminis/sakei* was found in batchs 20-I and 20-II (Figure 2B), however, the DNA sequence obtained from this band did not provide sufficient information to discriminate between these three *Lactobacillus* species even at 99% identity (Table 2). Differences were also found among sourdough batches fermented at 30°C. Fragments specific to *Lactobacillus brevis* and *P. pentosaceus* were seen only in sourdoughs 30-I and 30-III in addition to *L. plantarum*, which was found in all three batches. The DGGE pattern of 30-II had two additional bands, one of which was identified as *L. sakei* (Figure 2B and Table 2).

The diversity of the six bacterial communities continued to decrease during the second and third week of propagation (Figure 2B). On day 21, two major bands specific to the species *L. plantarum* and *L. brevis* were observed in sourdoughs 20-I and 20-III while *L. plantarum* and *Lactobacillus crustorum* were detected in sourdough 20-II. In sourdoughs fermented at 30°C, only one single strong band corresponding to either *Lactobacillus paralimentarius* (30-I) or *L. plantarum* (30-II and 30-III) was observed after cycle 21 (Figure 2B).

The bacterial composition of the sourdoughs was both stable and comparable between batches from day 21 to 35. However, further succession of species occurred after the fifth week of propagation (Figure 2C). On the final day of sampling, sourdoughs fermented at 20°C were comprised of either *L. brevis* and *L. paralimentarius* (sourdough 20-I), *L. brevis, L. plantarum* and *L. crustorum* (sourdough 20-II), or *L. brevis* and *L. plantarum* (sourdough 20-III). In sourdoughs fermented at 30°C *L. paralimentarius* was detected in all three sourdoughs either in combination with *L. plantarum* and *L. crustorum* (sourdough 30-I), together with *L. plantarum* (sourdough 30-II) or as a single dominant species (sourdough 30-III).

### Dynamics of the Bacterial Communities Determined by Pyrosequencing of Bar-coded 16S rRNA Gene Amplicons

Pyrosequencing of bar-coded 16S rRNA gene amplicons was applied to overcome limitation of DGGE analysis and study in-depth the establishment of microbial consortia in spontaneously started rye sourdoughs. Widely used V1–V2 region specific primers were chosen to distinguish between different LAB species [24]–[26]. After matching the barcodes and performing initial quality processing using PyroNoise, 48912 raw reads were found. After removing 1258 chimers and plant chloroplast related sequences, a total of 41819 high-quality partial 16S rRNA gene sequences longer than 150 bp were used in the analysis (Table S1). The majority of high-quality reads were in the length range 280–310 bp. The number of detected OTUs, normalized number of expected OTUs at 500 sequences, and the rate of new OTUs when one sequence is added to the sample at 500 sequences are provided in Table S1. The latter quantity indicates that the sequence coverage was sufficient for most of the samples, with the exception of four (0–20-I, 1–20-II, 1–30-I, 5–30-III), which all had a lower number of reads (≤500). During the first three propagation cycles, species diversity was higher in sourdoughs fermented at 30°C as determined by the normalized number of expected OTUs (Table S1). This number of expected OTUs per 500 reads declined during the continuous propagation of the sourdoughs to between 4 and 9 after 56 renewals (Table S1).

Several bacterial species were found in the rye flour (Table S2), although the ratio of microbial DNA to rye DNA in this sample was low. As a consequence, only 60 high-quality bacterial reads were obtained. The main OTUs identified were from the genera *Pantoea* (33%) and *Stenotrophomonas* (15%) (Table S2). LAB species were represented by *Lactobacillus iners* (3.3%), *Leuconostoc citreum* (1.7%), and *Weissella cibaria* (1.7%).

**Figure 3:**
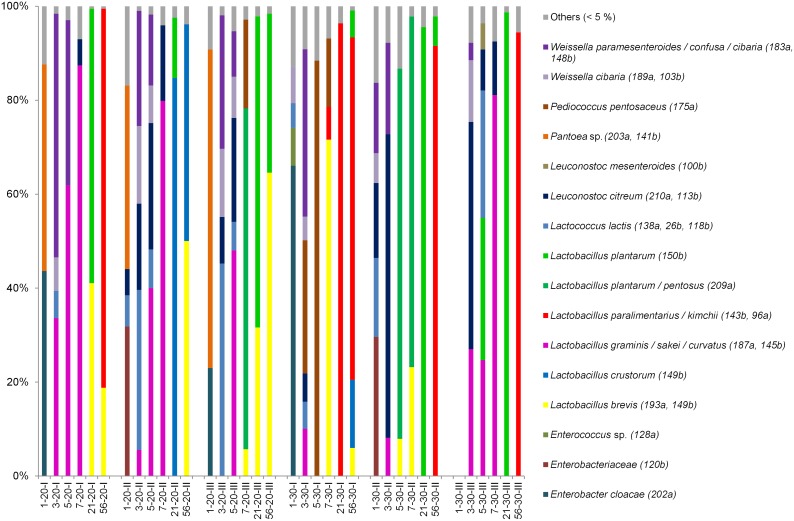
Pyrosequencing of 16S rRNA gene amplicons from spontaneous rye sourdoughs propagated for 56 days. Three sourdoughs were fermented at 20°C (20-I, 20-II, 20-III) and three sourdoughs fermented at 30°C (30-I, 30-II, 30-III). Sourdoughs were sampled at days 1, 3, 5, 7, 21, 56. The relative abundance at the species level based on partial 16S rRNA gene sequences is given. Species forming less than 5% of the population were grouped together and are shown as ‘Others (<5%)’. (a) and (b) stand for two different sequencing runs.

After 24 hours of spontaneous flour fermentation at 20°C between 70–90% of all identified 16S rRNA gene amplicons were from the genera *Enterobacter*, and *Pantoea* (Table S2). Bacteria from the genera *Weissella*, *Leuconostoc*, *Lactococcus,* and *Lactobacillus* together comprised up to 23% of the bacterial community. In sourdoughs fermented at 30°C, the majority of the bacterial community was formed by representatives of the genera *Enterobacter*, *Weissella*, *Lactococcus,* and *Leuconostoc*. In contrast with the DGGE analysis, differences between the bacterial composition of sourdoughs fermented under the same conditions were already observed after the first fermentation cycle (Figure 3).

In sourdoughs fermented at 20°C, enterobacteria were totally replaced by the LAB species Weissella cibaria, Weissella paramesenteroides/confusa, Lactococcus lactis, Leuconostoc citreum, and Lactobacillus graminis/sakei/curvatus after the third renewal cycle on day 3. W. paramesenteroides/confusa and Lc. lactis formed the dominant population in sourdoughs 20-II and 20-III. In batch 20-I, W. paramesenteroides/confusa and L. graminis/sakei/curvatus dominated. The proportion of L. graminis/sakei/curvatus increased in all sourdoughs fermented at 20°C after the fifth fermentation cycle. This species kept its dominant position in batches 20-I and 20-II up to day 8 and formed over 80% of the bacterial community. In contrast, L. plantarum/pentosus formed over 70% of the bacterial community in sourdough 20-III. After day 21, L. plantarum and L. brevis were the dominant species in sourdoughs 20-I and 20-III, while in sourdough 20-II L. crustorum and L. plantarum formed 85% and 13% of identified amplicons, respectively. Even after 56 days of fermentation the dominant community in all three sourdoughs fermented at 20°C differed. In sourdough 20-I, Lactobacillus paralimentarius/kimchii (the latter is the synonym for L. paralimentarius [27]) and L. brevis dominated in the proportion 81∶19. In sourdough 20-II the dominant community was represented by L. crustorum and L. brevis in the proportion of 46∶50. In sourdough 20-III L. plantarum and L. brevis were dominated in the proportion 34∶65.

In contrast with sourdoughs fermented at 20°C, enterobacteria were still present in low numbers within sourdoughs fermented at 30°C after the third renewal cycle. Various combinations of *W. paramesenteroides/confusa*, *Pediococcus pentosaceus*, *Leuc. citreum,* and *L. graminis/sakei/curvatus* were found in sourdoughs 30-I, 30-II, and 30-III. After the fifth renewal cycle, the dominant bacteria in 30-I and 30-II were *P. pentosaceus* and *L. plantarum/pentosus*, respectively. In sourdough 30-III, *L. plantarum*, *L. graminis/sakei/curvatus,* and *Lactococcus lactis* species were found in equal proportions (Figure 3). On day 7, over 70% of the bacterial community in sourdoughs 30-I, 30-II, and 30-III belonged to either *L. brevis, L. plantarum/pentosus,* or *L. graminis/sakei/curvatus*, respectively. However, on day 21 further changes in the composition of sourdoughs 30-I and 30-III were detected. Over 90% of the bacterial amplicons identified in sourdough 30-I belonged to *L. paralimentarius/kimchii,* while *L. plantarum* dominated in sourdoughs 30-II and 30-III. On day 56, *L. paralimentarius* was the dominant species (>70%) in all sourdoughs fermented at 30°C, while the proportion of *L. plantarum* and *L. brevis* remained below 7%. In sourdough 30-I, *L. crustorum* was also found to comprise 15% of the bacterial community after 56 backslopping cycles.

### Isolation, Identification, and Characterization of the Dominant Bacteria after 56 Backslopping Propagations

**Figure 4:**
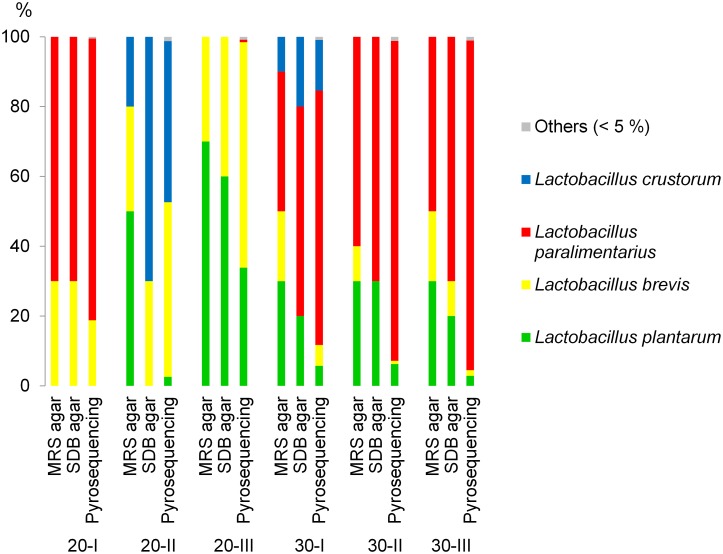
Ratio of species in the sourdoughs fermented at 20°C (20-I, 20-II, 20-III) or 30°C (30-I, 30-II, 30-III) after 56 backslopping cycles determined by plating on MRS and SDB media or by pyrosequencing of 16S rRNA gene amplicons.

In order to obtain pure cultures of bacteria after 56 days of propagation we randomly picked 10 colonies from MRS agar and 10 from SDB agar for each of the six samples. In total, 120 colonies were selected and analyzed by rep-PCR fingerprinting. Four different Rep-PCR fingerprint groups were detected (data not shown). Representatives of each group were identified using 16S rRNA partial gene sequencing and were found to be *L. plantarum* (*L. plantarum* M30I-1, GenBank accession number KJ361844), *L. brevis* (*L. brevis* M30I-2, GenBank accession number KJ361843), *L. paralimentarius* (*L. paralimentarius* M30I-3, Genbank accession number KJ361845) and *L. crustorum* (*L. crustorum* M30I-9, GenBank accession number KJ361846) with at least 98% identity. In most cases the fraction of each species identified in the sourdough samples are comparable with pyrosequencing data (Figure 4).

Metabolic profiles of the four dominant LAB species were determined (Table 3). The strain *L. plantarum* M30I-1 was able to ferment the largest range of carbohydrates. Together with *L. paralimentarius* M30I-3, *L. plantarum* M30I-1 was able to ferment all four major cereal carbohydrates (glucose, fructose, maltose, saccharose) while *L. brevis* M30I-2 and *L. crustorum* M30I-9 were able to ferment either glucose and fructose or glucose, fructose, and maltose, respectively.

**Table 3:** Carbohydrate fermentation profiles of *Lactobacillus* species isolated on day 56 of sourdough backlopping.

Active ingredient	*Lactobacillus* *plantarum*M30I-1	*Lactobacillus* *brevis*M30I-2	*Lactobacillus* *paralimentarius*M30I-3	*Lactobacillus* *crustorum*M30I-9
L-arabinose	+	+	+	−
D-ribose	+	+	+	−
D-xylose	+	+	+	−
D-galactose	+	+	−	+
D-glucose	+	+	+	+
D-fructose	+	+	+	+
D-mannose	+	−	+	+
D-mannitol	+	−	−	−
D-sorbitol	+	−	−	−
N-acetylglucosamine	+	+	+	+
Amygdalin	+	−	+	+
Arbutin	+	−	+	−
Esculin ferric citrate	+	−	+	−
Salicin	+	−	+	+
D-celiobinose	+	−	+	+
D-maltose	+	+	+	+
D-lactose (bovine origin)	+	−	−	+
D-melibiose	+	+	−	−
D-saccharose (sucrose)	+	−	+	−
D-trehalose	+	−	+	+
Inulin	+	−	−	−
D-melezitose	+	−	+	−
D-raffinose	+	−	−	−
Gentiobinose	+	−	+	+
Sodium Gluconate	+	+	+	−
Sodium 5-Ketogluconate	−	+	−	−

### Isolation, Identification, and Characterization of the Dominant Yeasts after 56 Backslopping Propagations

After 56 days of propagation 10 colonies were picked from YPD agar plates for each sourdough sample (60 colonies in total) and fingerprinted using M13 primer. Four different patterns were observed (data not shown) and representatives from each group were identified. They belong to *Kazachstania unispora* (*K. unispora* Y30I-10, GenBank accession number KJ361847), *Candida glabrata* (*C. glabrata* Y30II-1, GenBank accession number KJ361850), *Saccharomyces cerevisiae* (*S. cerevisiae* Y30II-9, GenBank accession number KJ361848) or *Candida krusei* (*C. krusei* Y30II-5, GenBank accession number KJ361849) based on 26S rRNA partial gene sequencing. *K. unispora* was found to be the only dominant species in all sourdoughs fermented at 20°C, but was also found in sourdough 30-I which was propagated at 30°C. *C. glabrata* was the only yeast species found in sourdough 30-III while sourdough 30-II contained three yeast species, *S. cerevisiae*, *C. krusei,* and *C. glabrata*, in the proportion 2∶3∶5.

Isolated representatives of *K. unispora, C. krusei,* and *C. glabrata* were not able to ferment maltose and had a narrow carbohydrate fermentation profile compared with *S. cerevisiae* (Table 4). However, only *C. krusei* and *C. glabrata* could ferment *N*-acetylglucosamine and trehalose, respectively.

**Table 4:** Carbohydrate fermentation profiles of yeast species isolated on day 56 of sourdough backlopping.

Active ingredient	*Saccharomyces* *cerevisiae*Y30II-9	*Kazachstania* *unispora*Y30I-10	*Candida* *krusei*Y30II-5	*Candida* *glabrata*Y30II-1
D-glucose	+	+	+	+
Glycerol	−	−	+	−
D-galactose	+	+	−	−
N-acetylglucosamine	−	−	+	−
D-maltose	+	−	−	−
D-saccharose (sucrose)	+	−	−	−
D-trehalose	−	−	−	+
D-melezitose	+	−	−	−
D-raffinose	+	−	−	−

## Discussion

Previous research has established that stabilization of LAB consortia in spontaneously started sourdoughs occurs in a three-stage evolution process over the course of five to ten days [2], [8], [10]–[11], [28]. During this time the acidity drops and stabilizes together with the LAB count to a level common in mature sourdough. The majority of these studies used liquid sourdoughs, which were fermented using continuous stirring. To our knowledge there are no studies that have monitored the fate of a bacterial community after the sourdough has reached maturity under controlled laboratory conditions, where the raw flour is the sole bacterial source.

We followed 56 daily backslopping cycles of spontaneously started semi-solid rye sourdough at two temperatures with six parallels. While the sourdoughs achieved maturity in 10 to 12 propagation cycles, further succession of LAB species was observed even after 42 cycles.

The low number of sourdough specific LAB in rye flour could be the main reason for the observed instability. The concentration of bacteria in rye flour varies between 10^4^ to 10^6^ CFU/g depending on the climate, time of harvest, and both milling and storage conditions [29]. Using 16S rRNA gene pyrosequencing we found that the microbiota in the raw flour used in this study contained LAB species at a subdominant level (<7%) and the majority of these were species that typically do not dominate in sourdough communities. Thus the strains that dominated in the six mature sourdough parallels may have been present in very low concentrations in the raw flour. The effect of introducing low numbers of sourdough competent LAB together with the small volumes of flour used to prepare the sourdoughs could together work to create an uneven distribution of bacteria between the three parallels conducted at the same temperature. As with our observations, Minervini et al. [31] observed the succession of LAB strains during propagation by comparatively following the microbial community of mature wheat sourdough during propagation in an artisan bakery and a controlled laboratory environment. They attribute the cause of the observed succession to be differences between the batches of flour used.

The temperature used for sourdough fermentation is one of the key factors determining the composition of the microbial community in sourdough [15], [32]. The process of establishing sourdough microbial consortia occurred more rapidly at 30°C, as evidenced by the prevalence of LAB in these sourdoughs already after the first fermentation cycle. Several studies have shown that only one fermentation at 30°C is needed to enrich LAB in sourdough [11], [32], whereas up to three renewal cycles were needed to detect LAB at a lower temperature (23°C) [32]. We also observed LAB after one fermentation at 20°C, albeit at a significantly lower fraction of the total community compared with fermentations at 30°C. In addition, the normalized number of expected OTUs indicates that the species richness during the first three propagation cycles was higher in sourdoughs fermented at higher temperature. Interestingly, enterobacteria persisted over more fermentation cycles in bacterial communities of sourdough fermented at 30°C (up to three fermentations) than in sourdoughs fermented at 20°C.

Facultative heterofermentative bacteria dominated in sourdoughs fermented at 30°C after 56 propagation cycles, while in sourdoughs fermented at 20°C both obligate and facultative heterofermentative LAB were dominant. It has been shown that despite the metabolic inefficiency of obligatory heterofermentative lactic acid bacteria, these species commonly dominate in sourdough fermentations [2]. Balance between homofermentative and heterofermentative lactic acid bacteria depends on dough yield, redox potential and fermentation temperature [30]. In this study fermentation temperature shifted the ratios between the limited number of LAB species in the community rather than select for different species. Rep-PCR analysis of isolates obtained from the sourdoughs after 56 renewal cycles revealed that fingerprints among isolates of the same species were similar.

Pyrosequencing results establish that during the development of bacterial consortia in sourdough, irrespective of fermentation temperature, *L. plantarum* dominated or codominated in the majority of sourdoughs. However, after 56 propagation cycles *L. plantarum* had been replaced by *L. paralimentarius* in most batches. *L. plantarum* is considered a highly acid-tolerant LAB that dominates in fermentation processes with vegetables and cereals due to its metabolic flexibility and low pH adaptation [33]–[35]. It also dominated in bacterial communities of four liquid spontaneously started laboratory rye sourdoughs after 10 backslopping renewals [8]. The carbohydrate fermentation pattern of *L. paralimentarius* isolated in this study is more constrained compared with *L. plantarum,* however, they are able to ferment all major carbohydrates (maltose, saccharose, fructose, and glucose) [36]. On the other hand, succession within bacterial communities may depend on many other factors aside from carbohydrate metabolism, including amino acid metabolism and tolerance to acid stress conditions [10]. Several studies have shown that the competiveness of LAB often depends on their intraspecies diversity and is strain-specific [28], [36].

Fermentation temperature also influenced the composition and diversity of the yeast community within the sourdoughs studied. Both yeast species detected in 30°C fermentations after 56 propagation cycles, i.e. *Saccharomyces cerevisiae* and *Candida krusei* belong to the six most frequently encountered species in sourdoughs [37], while *Candida glabrata* is considered to be a prevailing species during liquid laboratory sourdough and teff fermentations [28], [38]. *Kazachstania unispora* was the only yeast species found in sourdoughs fermented at 20°C. This species has been documented twice to exist in a sourdough ecosystem, albeit in low abundance: once in Belgian artisan wheat sourdough [37] and once in Finnish rye sourdough starter [39]. In contrast, *K. unispora* plays a significant role in both the ripening of cheese and in the production of fermented milk products such as a kefir and koumiss (reviewed by [40]). Additionally orange, sugarcane or mixed vegetable juices favour growth of *K. unispora*. Ambient or low temperatures and high organic acid concentrations are specific for most of these processes [38]–[39], [41]–[44]. No clear relationship was found between the yeast and LAB strains detected in this study. However, most isolated yeast species were maltose negative and trophic interactions between LAB and yeasts could be suspected because cooperation in utilization of maltose is the most frequently cited reason for the co-occurrence of yeasts and LAB (reviewed in [45]).

Studies of microbial consortia in food matrices, such as sourdoughs, are commonly based on a combination of culture dependent and culture independent methods [46]–[47]. This study applies both approaches to analyze the bacterial community within mature sourdoughs after 56 propagation cycles. Plating on both SDB and MRS media, which are both commonly used for sourdough specific LAB isolation [48], provided similar numbers of colony forming units. In most samples species occurrence and ratio among identified isolates was comparable with pyrosequencing data.

The results of both DGGE and 16S rRNA gene pyrosequencing were generally in agreement despite the fact that they amplified different regions of the 16S rRNA. The bacterial diversity suggested by pyrosequencing results was greater than that revealed by DGGE because of the differences in their detection limits. Both methods are based on DNA amplification and share similar limitations, e.g. possible inefficient DNA isolation from some organisms. However, high-throughput sequencing analysis is considered quantitative if efforts are made to minimize changes in the original proportion of microbial cells caused by DNA extraction [11]. Amplification of DNA from dead organisms could be critical for the first step of sourdough propagation which would overestimate the diversity of metabolically active bacteria in flour and sourdoughs. Similar distribution of OTUs between DNA and RNA samples has been shown for mature sourdoughs [11].

Both DGGE and pyrosequencing often fail to discriminate between closely related species due to both the insufficient length and accuracy of sequences. Current developments in high-throughput sequencing technologies are expected to overcome these problems. Despite its limitations, high-throughput sequencing has the potential to become a powerful tool for the culture-independent study of sourdough microflora since it offers a more in-depth analysis [49].

## Supporting Information

Table S1 Characterization of pyrosequencing data obtained from the analysis of sourdough samples.
(PDF)

Table S2 Relative abundance (%) of partial 16S rRNA gene sequences obtained by pyrosequencing of spontaneous rye sourdoughs propagated for 56 days.
(PDF)

## References

[1] Gobbetti M, Simonetti MS, Corsetti A, Santinelli F, Rossi, P at al (1995) Volatile compound and organic acid production by mixed wheat sour dough starters: influence of fermentation parameters and dynamics during baking. Food Microbiology 12: 497–507.

[2] De Vuyst L, Neysens P (2005) The sourdough microflora: biodiversity and metabolic interactions. Trends Food Sci Technol 16: 43–56.

[3] Diowksz A, Ambroziak W (2006) Sourdough. In: Hui YH, Corke H, De Leyn I, Nip WK, Cross NA, editors. Bakery Products: Science and Technology. Oxford: Blackwell Publishing Ltd. 365–380.

[4] Rosenquist H, Hansen A (2000) The microbial stability of two bakery sourdoughs made from conventionally and organically grown rye. Food Microbiol 17: 241–250.

[5] Hansen AS (2004) Sourdough bread. In: Hui YH, Meunier-Goddik L, Hansen AS, Josephsen J, Nip WK et al.., editors. Handbook of Food and Beverage Fermentation Technology. New York: Marcel Dekker, Inc. 840–870.

[6] Müller MRA, Wolfrum G, Stolz P, Ehrmann MA, Vogel RF (2001) Monitoring the growth of *Lactobacillus* species during a rye flour fermentation. Food Microbiol. 18: 217–227.

[7] Vogelmann SA, Seitter M, Singer U, Brandt MJ, Hertel C (2009) Adaptability of lactic acid bacteria and yeasts to sourdoughs prepared from cereals, pseudocereals and cassava and use of competitive strains as starters. Int J Food Microbiol 130: 205–212. 19239979 10.1016/j.ijfoodmicro.2009.01.020

[8] Weckx S, Van der Meulen R, Maes D, Scheirlinck I, Huys G, et al (2010) Lactic acid bacterial community dynamics and metabolite production of rye sourdough fermentations share characteristics of wheat and spelt sourdough fermentations. Food Microbiol 27: 1000–1008. 20832677 10.1016/j.fm.2010.06.005

[9] Viiard E, Mihhalevski A, Rühka T, Paalme T, Sarand I (2013) Evaluation of the microbial community in industrial rye sourdough upon continuous back–slopping propagation revealed *Lactobacillus helveticus* as the dominant species. J Appl Microbiol 114: 404–12. 23082800 10.1111/jam.12045

[10] Van der Meulen R, Scheirlinck I, Van Schoor A, Huys G, Vancanneyt M, et al (2007) Population dynamics and metabolite target analysis of lactic acid bacteria during laboratory fermentations of wheat and spelt sourdoughs. Appl Environ Microbiol 73: 4741–4750. 17557853 10.1128/AEM.00315-07 PMC1951026

[11] Ercolini D, Pontonio E, De Filippis F, Minervini F, La Storia A, et al (2013) Microbial ecology dynamics during rye and wheat sourdough preparation. Applied and Environmental Microbiology 79(24): 7827–36. 24096427 10.1128/AEM.02955-13 PMC3837820

[12] Hammes WP, Stolz P, Gänzle M (1996) Metabolism of lactobacilli in traditional sourdoughs. Adv Food Sci 18: 176–184.

[13] Hammes WP, Gänzle MG (1998) Sourdough breads and related products. In: Woods BJB, editor. Microbiology of Fermented Foods, vol 1. London: Blackie Academic. 199–216.

[14] Gobbetti M (1998) The sourdough microflora: interactions of lactic acid bacteria and yeasts. Trends food Sci Technol 9: 267. 10.1007/BF00414862 24421010

[15] Meroth CB, Walter J, Hertel C, Brandt MJ, Hammes WP (2003) Monitoring the bacterial population dynamics in sourdough fermentation processes by using PCR-denaturing gradient gel electrophoresis. Appl Environ Microbiol 69: 474–482. 10.1128/AEM.69.1.475-482.2003 PMC152404 12514030

[16] Wick M, Stolz P, Böcker G, Lebault JM (2003) Influence of several process parameters on sourdough fermentation. Acta Biotechnol 23: 51–61.

[17] McKenna P, Hoffmann C, Minkah N, Aye PP, Lackner A, et al (2008) The macaque gut microbiome in health, lentiviral infection, and chronic enterocolitis. PLoS Pathog 4(2): 20. 10.1371/journal.ppat.0040020 PMC2222957 18248093

[18] Schloss PD, Westcott SL, Ryabin T, Hall JR, Hartmann M, et al (2009) Introducing mothur: open-source, platform-independent, community-supported software for describing and comparing microbial communities. Appl Environ Microbiol 75: 7537–41. 19801464 10.1128/AEM.01541-09 PMC2786419

[19] Pruesse E, Quast C, Knittel K, Fuchs BM, Ludwig W, et al (2007) SILVA: a comprehensive online resource for quality checked and aligned ribosomal RNA sequence data compatible with ARB. Nucleic Acids Res 35: 7188–7196. 17947321 10.1093/nar/gkm864 PMC2175337

[20] Andrighetto C, Psomas E, Tzanetakis N, Suzzi G, Lombardi A (2000) Randomly amplified polymorphic DNA (RAPD) PCR for the identification of yeasts isolated from dairy products. Letters in Applied Microbiology 30(1): 5–9. 10728551 10.1046/j.1472-765x.2000.00589.x

[21] Frank JA, Reich CI, Sharma S, Weisbaum JS, Wilson BA, et al (2008) Critical evaluation of two primers commonly used for amplification of bacterial 16S rRNA genes. Appl Environ Microbiol 74: 2461–2470. 18296538 10.1128/AEM.02272-07 PMC2293150

[22] Weisburg WG, Barns SM, Pelletier DA, Lane DJ (1991) Ribosomal DNA amplification for phylogenetic study. J Bacteriol 173: 697–703. 1987160 10.1128/jb.173.2.697-703.1991 PMC207061

[23] Siti Hajar MD, Noorhisham TK, Nurina A (2012) Short Technical Communication Yeast identification from domestic ragi for food fermentation by PCR method. International Food Research Journal 19(2): 775–777.

[24] Balcazar JL, de Blas I, Ruiz-Zarzuela I, Vendrell D, Girones O, et al (2007) Sequencing of variable regions of lactic acid bacteria isolated from the intestinal microbiota of healthy salmonids. Comparative Immunology, Microbiology and Infectious Diseases 30: 111–118. 10.1016/j.cimid.2006.12.001 17239438

[25] Huse SM, Ye Y, Zhou Y, Fodor A (2012) A core human microbiome as viewed through 16S rRNA sequence clusters. PloS One 7(6): e34242 doi:10.1371/journal.pone.0034242 22719824 10.1371/journal.pone.0034242 PMC3374614

[26] Delgado S, Rachid CT, Fernandez E, Rychlik T, Alegria A, et al (2013) Diversity of thermophilic bacteria in raw, pasteurized and selectively-cultured milk, as assessed by culturing, PCR-DGGE and pyrosequencing. Food Microbiol 36: 103–111. 23764225 10.1016/j.fm.2013.04.015

[27] Pang H, Kitahara M, Tan Z, Wang Y, Qin G, et al (2012) Reclassification of Lactobacillus kimchii and Lactobacillus bobalius as later subjective synonyms of Lactobacillus paralimentarius. Int J Syst Evol Microbiol 62: 2383–2387. 22140166 10.1099/ijs.0.035329-0

[28] Moroni AV, Arendt EK, Dal Bello F (2011) Biodiversity of lactic acid bacteria and yeasts in spontaneously-fermented buckwheat and teff sourdoughs. Food Microbiol 28: 497–502. 21356457 10.1016/j.fm.2010.10.016

[29] Lonner C (1988) Starter Cultures for Rye Sour Dough, Ph.D. Dissertation. Lund University, Sweden.

[30] Minervini F, De Angelis M, Di Cagno R, Gobbetti M (2014) Ecological parameters influencing microbial diversity and stability of traditional sourdough. International Journal of Food Microbiology 171: 136–146. 24355817 10.1016/j.ijfoodmicro.2013.11.021

[31] Minervini F, Lattanzi A, De Angelis M, Di Cagno R, Gobbetti M (2012) Influence of Artisan Bakery- or Laboratory-Propagated Sourdoughs on the Diversity of Lactic Acid Bacterium and Yeast Microbiotas. Applied and Environmental Microbiology 78(15): 5328–5340. 22635989 10.1128/AEM.00572-12 PMC3416412

[32] Vrancken G, Rimaux T, Weckx S, Leroy F, De Vuyst L (2011) Influence of temperature and backslopping time on the microbiota of a type I propagated laboratory wheat sourdough fermentation. Applied and Environmental Microbiology 77(8): 2716–26. 21335386 10.1128/AEM.02470-10 PMC3126363

[33] Vrancken G, De Vuyst L, Rimaux T, Allemeersch J, Weckx S (2011) Adaptation of *Lactobacillus plantarum* IMDO 130201, a wheat sourdoughisolate, to growth in wheat sourdough simulation medium at different pH values through differential gene expression. Appl Environ Microbiol 77: 3406–12. 21460118 10.1128/AEM.02668-10 PMC3126451

[34] Boekhorst J, Siezen RJ, Zwahlen MC, Vilanova D, Pridmore RD, et al (2009) The complete genomes of Lactobacillus plantarum and Lactobacillus johnsonii reveal extensive differences in chromosome organization and gene content. Microbiology 150: 3601–3611. 10.1099/mic.0.27392-0 15528649

[35] Plumed-Ferrer C, Koistinen KM, Tolonen TL, Lehesranta SJ, Kärenlampi SO, et al (2008) Comparative study of sugar fermentation and protein expression patterns of two Lactobacillus plantarum strains grown in three different media. Appl Environ Microbiol 74: 5349–5358. 18567686 10.1128/AEM.00324-08 PMC2546631

[36] Minervini F, De Angelis M, Di Cagno R, Pinto D, Siragusa S, et al (2010) Robustness of Lactobacillus plantarum starters during daily propagation of wheat flour sourdough type I. Food Microbiol. 27: 897–908. 10.1016/j.fm.2010.05.021 20688231

[37] Huys G, Daniel HM, De Vuyst L (2013) Taxonomy and Biodiversity of Sourdough Yeasts and Lactic Acid Bacteria. In: Gobbetti M, Gänzle M, editors. Handbook on Sourdough Technology. New York: Springer Science+Business Media. 107.

[38] Vrancken G, De Vuyst L, Van der Meulen R, Huys G, Vandamme P, et al (2010) Yeast species composition differs between artisan bakery and spontaneous laboratory sourdoughs. FEMS Yeast Res 10(4): 471–81. 20384785 10.1111/j.1567-1364.2010.00621.x

[39] Salovaara H, Savolainen J (1984) Yeast types isolated from Finnish sourdough starters. Acta Alimentaria Polonica 10: 242–246.

[40] Bhattacharya I, Yan S, Yadav JSS, Tyagi RD, Surampalli RY (2013) *Saccharomyces unisporus*: Biotechnological Potential and Present Status. Comprehensive Reviews in Food Science and Food Safety 12(4): 353–363. 10.1111/1541-4337.12016 33412685

[41] Savard T, Beaulieu C, Boucher I, Champagne CP (2002) Antimicrobial action of hydrolyzed chitosan against spoilage yeasts and lactic acid bacteria of fermented vegetables. Journal of Food Protection 65(5): 828–33. 12030295 10.4315/0362-028x-65.5.828

[42] Callon C, Delbès C, Duthoit F, Montel MC (2006) Application of SSCP-PCR fingerprinting to profile the yeast community in raw milk Salers cheeses. Systematic and Applied Microbiology 29(2): 172–80. 16464699 10.1016/j.syapm.2005.07.008

[43] Londero A, Hamet MF, De Antoni GL, Garrote GL, Abraham AG (2012) Kefir grains as a starter for whey fermentation at different temperatures: chemical and microbiological characterisation. Journal of Dairy Research 79(3): 262–71. 22717048 10.1017/S0022029912000179

[44] Mu Z, Yang X, Yuan H (2012) Detection and identification of wild yeast in Koumiss. Food Microbiology 31: 301–308. 22608237 10.1016/j.fm.2012.04.004

[45] Daniel HM, Moons MC, Huret S, Vrancken G, De Vuyst L (2011) *Wickerhamomyces anomalus* in the sourdough microbial ecosystem. Antonie Van Leeuwenhoek 99(1): 63–73. 20963492 10.1007/s10482-010-9517-2

[46] Temmerman R, Huys G, Swings J (2004) Identification of lactic acid bacteria: culture-dependent and culture -independent methods. Trends Food Sci Technol 15: 348–359.

[47] Cocolin L, Alessandria V, Dolci P, Gorra R, Rantsiou K (2013) Culture independent methods to assess the diversity and dynamics of microbiota during food fermentation. Int J Food Microbiol, http://dx.doi.org/10.1016/j.ijfoodmicro.2013.05.008. 10.1016/j.ijfoodmicro.2013.05.008 23791362

[48] Vera A, Rigobello V, Demarigny Y (2009) Comparative study of culture media used for sourdough lactobacilli. Food Microbiology 26(7): 728–33. 19747606 10.1016/j.fm.2009.07.010

[49] Ercolini D (2013) High-throughput sequencing and metagenomics: moving forward in the culture-independent analysis of food microbial ecology. Applied and Environmental Microbiology 79(10): 3148–55. 23475615 10.1128/AEM.00256-13 PMC3685257

