# Supplementary material for: Evolution of Bacterial Consortia in Spontaneously Started Rye Sourdoughs during Two Months of Daily Propagation
Source: PLoS One. 2014 Apr 18;9(4):e95449. doi: 10.1371/journal.pone.0095449 (PMC3991677; doi:10.1371/journal.pone.0095449)
Supplement: Table S1 — Characterization of pyrosequencing data obtained from the analysis of sourdough samples. (PDF) [file pone.0095449.s001.pdf]

Sample	Number of raw reads	Number of reads	Number of OTUs	Number of expected OTUs at 500 reads	Rate of new OTUs at 500 reads
0-20-I	833	60	21	21	NA*
1-20-I	5496	4488	42	15	0.012
1-20-II	607	556	34	32	0.038
1-20-III	3462	3262	29	16	0.012
1-30-I	291	286	20	20	NA
1-30-II	624	614	25	23	0.018
3-20-I	2060	1886	16	8	0.006
3-20-II	1787	1658	18	9	0.008
3-20-III	1487	1393	16	9	0,008
3-30-I	1591	1112	29	19	0.018
3-30-II	1921	1734	24	14	0.011
3-30-III	1755	1507	25	15	0.013
5-20-I	2019	1852	10	7	0.003
5-20-II	1779	1582	20	11	0.010
5-20-III	720	660	17	15	0.011
5-30-I	2376	1287	19	12	0.011
5-30-II	1595	1303	23	13	0.015
5-30-III	594	413	15	15	NA

7-20-I	1814	1639	13	8	0.005
7-20-II	1565	1377	17	11	0.009
7-20-III	1473	1086	19	12	0.014
7-30-I	1748	1472	22	13	0.014
7-30-II	1077	958	13	9	0.010
7-30-III	1277	1163	12	9	0.006
21-20-I	621	565	5	5	0.005
21-20-II	870	813	4	4	0.001
21-20-III	726	645	9	8	0.008
21-30-I	664	607	7	6	0.007
21-30-II	790	766	12	9	0.013
21-30-III	705	700	10	7	0.013
56-20-I	596	575	4	4	0.002
56-20-II	758	728	11	9	0.010
56-20-III	765	706	8	7	0.007
56-30-I	818	786	11	8	0.009
56-30-II	759	737	11	9	0.010
56-30-III	889	843	11	8	0.009
Total	48912	41819			

* NA, not analyzed since the number of obtained reads was less than 500.
