# Supplementary material for: Evolution of Bacterial Consortia in Spontaneously Started Rye Sourdoughs during Two Months of Daily Propagation
Source: PLoS One. 2014 Apr 18;9(4):e95449. doi: 10.1371/journal.pone.0095449 (PMC3991677; doi:10.1371/journal.pone.0095449)
Supplement: Table S2 — Relative abundance (%) of partial 16S rRNA gene sequences obtained by pyrosequencing of spontaneous rye sourdoughs propagated for 56 days. (PDF) [file pone.0095449.s002.pdf]

Closest match	OTU	Accession number	0-20-I	1-20-I	1-20-II	1-20-III	1-30-I	1-30-II	3-20-I	3-20-II	3-20-III	3-30-I	3-30-II	3-30-III	5-20-I	5-20-II	5-20-III	5-30-I	5-30-II	5-30-III	7-20-I	7-20-II	7-20-III	7-30-I	7-30-II	7-30-III	21-20-I	21-20-II	21-20-III	21-30-I	21-30-II	21-30-III	56-20-I	56-20-II	56-20-III	56-30-I	56-30-II	56-30-III
Burkholderia sp.	67b*	KJ127807	1.7																																			
Chryseobacterium sp.	79b	KJ127816	1.7																																			
Clostridium perfringens	124a*	KJ127663					0.7																															
Curtobacterium sp.	89b	KJ127821	1.7																																			
Curtobacterium sp.	83b	KJ127818	1.7																																			
Enterobacter cloacae	202a	KJ127739		43.6		23.0	66.1					4.6		0.7																								
Enterobacteriaceae	120b	KJ127849			31.8			29.6																														
Enterobacteriaceae	205a	KJ127742		1.4																																		
Enterobacteriaceae	105b	KJ127834			1.1																																	
Enterobacteriaceae	116b	KJ127845	1.7																																			
Enterococcus faecium	117b	KJ127846						2.3																														
Enterococcus hermanniensis	114b	KJ127843			2.2			1.3																														
Enterococcus hermanniensis	188a	KJ127725					1.0																															
Enterococcus sp.	128a	KJ127666					8.0					1.4																										
Erwinia sp.	201a	KJ127738		0.6		2.3	0.7																															
Frigoribacterium sp.	94b	KJ127823	5.0																																			
Lactobacillus brevis	149b	KJ127876																		1.0							41.1	2.3	31.6	1.5	3.0		18.8	50.0	64.6	6.0	0.9	1.7
Lactobacillus brevis	193a	KJ127730																5.0	7.9		0.9		5.7	71.6	23.2	0.7												
Lactobacillus crustorum	140b	KJ127869																										84.7						46.2		14.5		
Lactobacillus crustorum	208a	KJ127745																				1.0																
Lactobacillus graminis / sakei / curvatus	187a	KJ127724		0.7			1.4		33.6	5.5	1.1	10.1	8.1	27.0	61.9	40.0		2.1	4.7		87.4	79.9				81.1												
Lactobacillus graminis / sakei / curvatus	145b	KJ127872			1.4			1.6									48.0			24.7									1.1									
Lactobacillus iners	2b	KJ127750	3.3																																			
Lactobacillus paralimentarius / kimchii	143b	KJ127871																												96.4			80.7		0.7	72.9	91.6	94.4
Lactobacillus paralimentarius / kimchii	96a	KJ127638																1.5						7.0														
Lactobacillus plantarum	156a	KJ127694																					0.9															
Lactobacillus plantarum / pentosus	209a	KJ127746										0.8	1.8	0.7				1.6	78.8			0.8	72.6	4.5	74.6	4.0												
Lactobacillus plantarum	150b	KJ127877															1.1			30.3							58.4	12.8	66.2	1.5	95.6	98.7		2.6	33.9	5.7	6.2	2.8
Lactococcus lactis	138a	KJ127676				1.9	5.2		5.8	34.1	45.2	5.8	0.9	1.7		8.2																						
Lactococcus lactis	118b	KJ127847			6.7			16.8									6.1																					
Lactococcus lactis	26b	KJ127770															1.2			27.1																		
Lactococcus sp. / *garvieae* / *Edwardsiella tarda*	102b	KJ127831						1.0																														
Leuconostoc citreum	113b	KJ127842	1.7		5.6			16.0									22.1			8.7																		
Leuconostoc citreum	210a	KJ127747				0.6	0.7		1.0	18.3	9.9	6.0	64.6	48.4	0.7	26.9			3.5		5.6	16.0				11.4												
Leuconostoc mesenteroides	100b	KJ127829						3.6									1.2			5.6																		
Luteibacter sp. / Xanthomonadaceae bacterium	92b	KJ127822	3.3																																			
Paenibacillus sp.	121b	KJ127850	6.7																																			
Pantoea agglomerans	149a	KJ127687		0.6		1.1																																
Pantoea sp. /agglomerans / ananatis	204a	KJ127741		2.5																																		
Pantoea sp.	141b	KJ127870	33.3		39.0			3.4																														
Pantoea sp. / *agglomerans / vagans*	203a	KJ127740		44.0		67.8	0.7																															
Pediococcus pentosaceus	175a	KJ127713										28.3		3.2		0.6		88.4	3.1		2.3	0.8	18.9	14.5	0.9	1.5												
Pedobacter	71b	KJ127810	1.7																																			
Pedobacter	76b	KJ127815	1.7																																			
Pseudomonas marginalis / sp.	101b	KJ127830	3.3																																			
Rahnella aquatilis	190a	KJ127727		0.6																																		
Rhizobiaceae bacterium	80b	KJ127817	6.7																																			
Rhizobium sp.	1b	KJ127749	3.3																																			
Staphylococcus sp. / *gallinarum / succinus*	86b	KJ127820	1.7																																			
Stenotrophomonas sp.	115b	KJ127844	15.0																																			
Stenotrophomonas sp.	69b	KJ127808	1.7																																			
Stenotrophomonas sp.	84b	KJ127819	1.7																																			
Weissella cibaria	103b	KJ127832	1.7		3.4			6.4									8.8																					
Weissella cibaria	189a	KJ127726				0.5	7.7		7.1	16.5	14.5	5.0	2.4	13.1	1.2	8.0																						
Weissella paramesenteroides / confusa	183a	KJ127720		4.4		0.7	4.5		51.9	24.5	28.4	35.6	19.5	3.7	35.2	15.1					3.3																	
Weissella paramesenteroides / confusa / cibaria	148b	KJ127875			3.4			15.0									9.7																					
Others (< 0.5 %)				1.5	5.4	2.1	3.1	3.1	0.6	1.0	0.9	2.3	2.6	1.5	1.0	1.2	1.8	1.5	1.9	2.7	0.6	1.5	1.9	2.4	1.3	1.3	0.5	0.1	1.1	0.7	1.4	1.3	0.5	1.2	0.8	0.9	1.2	1.1

* (a) and (b) stand for two different sequencing runs.
